# Validation of the Parent-report Pandemic Anxiety Scale (PAS-P) in the context of COVID-19

**DOI:** 10.1007/s12144-024-06784-x

**Published:** 2024-10-10

**Authors:** Olly Robertson, Simona Skripkauskaite, Cathy Creswell, Eoin McElroy, Polly Waite

**Affiliations:** 1 Department of Psychiatry, University of Oxford, Oxford, UK; 2 Department of Experimental Psychology, University of Oxford, Anna Watts Building, Radcliffe Observatory Quarter, Woodstock Road, Oxford, OX2 6GG UK; 3 School of Psychology, Ulster University, Coleraine, UK

**Keywords:** COVID-19 pandemic, Anxiety, Child, Parent, PAS-P, UK

## Abstract

To be able to develop effective policy and targeted support for children and young people, it is vital to develop and validate measures that enable us to understand what aspects of pandemics are associated with anxiety and stress across a wide age range. We examined the psychometric properties of the Pandemic Anxiety Scale– Parent-report (PAS-P), which measures levels of child and adolescent pandemic-related anxieties. Factor structure, reliability, and convergent and discriminant validity of the PAS-P was assessed in a convenience sample of parents/carers (*N­ *= 8410) over at three time points in 2020 when COVID-19 case rates and restrictions varied. Factor structure was identified via two exploratory factor analyses (EFAs; *n *= 5601 and *n *= 1005) and then tested using confirmatory factor analysis (CFA; *n *= 800), measurement invariance tests, and a longitudinal CFA (*n *= 1651). Two factors structure for disease and consequence anxiety were observed across both EFAs and were found to have good fit in the CFAs. The PAS-P demonstrated good internal consistency and discriminant validity, as well as partial scalar invariance in latent construct measurement across child age, gender, and time. The PAS-P is a robust parent-report measure of two distinct forms of pandemic-related anxiety, suitable for reporting on children and adolescents aged 4-16 years. Although the scale has been validated in the context of the COVID-19 pandemic, it is not limited to this specific pandemic and, with minor wording modifications, may be a reliable tool in other health epidemic contexts.

**Supplementary Information:**

The online version contains supplementary material available at 10.1007/s12144-024-06784-x.

## Introduction

The public health response to the COVID-19 pandemic is likely to have had negative consequences for the mental health and wellbeing of children and adolescents (Newlove-Delgado et al., 2023). Negative mental health outcomes have been attributed to increased social isolation due to school closures and restrictions, increased stress over home-schooling expectations, and the cancellation of milestone events (e.g., O’Sullivan et al., 2021; Pearcey et al., 2023). It is also likely that increased mental health difficulties have related to concerns around the disease itself (e.g., getting COVID-19 and passing it onto others, particularly those who are vulnerable; Taylor et al., 2020). To be able to develop effective policy and targeted support for children and young people in the future, it is vital to develop and validate measures that enable us to understand what aspects of pandemics appear to be associated with anxiety and stress. Furthermore, changes in children and young people’s mental health during the COVID-19 pandemic has differed according to demographic characteristics, such as child age and gender (Creswell et al., 2021b; Guzman Holst et al., 2023). So, any measure of pandemic-related anxieties must be validated across these groups to ensure the construct of interest is assessed robustly.

The Pandemic Anxiety Scale (PAS; McElroy et al., 2020) is a brief, validated self-report measure that was developed to measure worries related to specific aspects or contextual factors in pandemics. It contains seven items capturing worries about the disease itself, such as catching or transmitting the disease, and the long-term outcomes associated with the public health response, such as future economic uncertainty. Early in the COVID-19 pandemic (March-April 2020), McElroy and colleagues (2020) examined the psychometric properties of the scale in a sample of adults (aged 18–71 years) and adolescents (aged 11–17 years). In the adolescent sample, they found that female and older participants were more likely to self-report concerns about the long-term consequences of the pandemic than male and younger adolescents. Gender differences in their findings could potentially be explained by the more general pre-pandemic gender inequality in young people’s mental health with girls reporting higher psychological distress and more negative feeling than boys (Hartas, 2021, 2023). Yet, it is likely that with the closure of schools, cancellation of national examinations and shutting down of large parts of the economy during the first national lockdown, the educational and economic impact were most directly relevant to the older adolescents.

Any measure of pandemic-related anxiety must be able to capture the experiences of children and young people across a large age range, including younger children who may not be able to provide reports themselves. In such cases, parents and carers, as main caregivers, are likely to be an excellent source of information (Pickard & Knight, 2005). Given the low-to-moderate agreement between child and parent responses for anxiety measures (e.g., Barbosa et al., 2002; van der Meer et al., 2008), it is best practice in the assessment of child anxiety to obtain reports from both the child and a parent/carer where possible (Silverman & Ollendick, 2008) but to prioritize the parent/carer report for preadolescent children (Creswell et al., 2021). Despite the robust validation of the self-report PAS in an adolescent (McElroy et al., 2020) and adult populations (Kubb & Foran, 2020; McElroy et al., 2020), a parent-report version of the PAS has not yet been validated. Indeed, to our knowledge, to date there is no validated parent/carer report measure which captures pandemic-related anxieties in children and adolescents.

Pandemic-related anxiety measures must also be able to capture the likely differences in pandemic-related worry at different time points across a pandemic and thus longitudinal assessment of measurement model stability is necessary. At least on average, children and young people’s mental health appears to have worsened during national lockdowns and at times of high restrictions, and improved when restrictions eased (Barendse et al., 2023; Creswell et al., 2021b). Yet, these changes in circumstances could have affected not only the amount of the pandemic-related worry but also how the pandemic-related concerns were perceived, thus reducing the longitudinal validity of a measure.

The aim of the present study was to validate the parent/carer-report version of the PAS (PAS-P) that was completed during the COVID-19 pandemic. Through validating the PAS-P, we hope to provide a quick and reliable method of measuring child and adolescent pandemic-related anxiety, which together with the young person’s self-reported PAS could provide a holistic overview of their psychological distress during the pandemic. The present psychometric investigation includes factor structure, reliability, and convergent and discriminant validity in a large sample of parent/carers, reporting on children and adolescents living in the UK. The current work also assesses measurement invariance across child age and gender, while using longitudinal data collected at three time points in 2020 when COVID-19 case rates and restrictions varied. We hypothesised that the factor structure obtained through the exploratory factor analysis would be supported by confirmatory factor analysis and remain invariant across time points and demographic groups.

## Method

### Participants and recruitment

All participants were recruited from the UK-based longitudinal Co-SPACE (COVID-19: Supporting Parents, Adolescents and Children during Epidemics) survey investigating the effects of the COVID-19 pandemic on the well-being of children, adolescents, and parents/carers (Waite et al., 2020). To be eligible to take part in the Co-SPACE study, participants had to be a parent or carer (henceforth known as ‘parents’) of a 4-16-year-old child and live in the UK. Participants were recruited through a variety of means, including promoting the study through partner organisations, networks, charities and schools, print and digital media coverage, and social media.

In total, data from 6,759 parents reporting on pandemic anxiety as experienced by their child were included in the present analyses (see Supporting Information: Table S1 for demographic information). To be included, participants had to have completed a survey during at least one of the three time periods of interest in 2020. These periods were selected based on the qualitatively varying national and local restrictions (Figure S1 in Supporting Information) that may affect factor structure invariance. Wave 1 (30th March to 29th May 2020) began shortly after the start of the national lockdown, when the public were required to stay at home except for very limited purposes and children were largely home-schooled. Wave 2 (1st July to 31st August 2020) was characterised by an overall easing of restrictions, including the phased reopening of schools in Scotland and Northern Ireland^1^, with regional lockdowns to manage high case numbers in specific areas. Wave 3 (1st November to 31st December 2020) was distinguished by a tightening of restrictions, tiered regional lockdowns in England and Scotland, and national lockdowns in Wales and Northern Ireland but schools remained largely open for face-to-face teaching during this period. Participants were also required to have completed all items on the PAS-P, as well as provided their child’s age and gender variables. In cases where child gender was not reported as female or male, participants were excluded from analyses of gender due to the small sample size (< 1% of the sample).

### Procedure

Ethical approval for the study was granted by the University’s Ethics Committee (reference R69060). Parents provided informed consent before proceeding to the survey, which was administered through Qualtrics. Parents of multi-child families were asked to identify one child who they would report on each time. Following completion of their first survey, participants were invited back monthly for a follow-up survey. From December 2020, participants were offered the chance to win a £50 voucher in return for their participation.

### Measures

#### Demographic variables

Parents reported their child’s age, gender, and ethnicity, and their own gender and ethnicity. Due to the typical differences in patterns of child and adolescent mental health and their different educational experiences, we dichotomised age at baseline as 4-10-year-olds (children) and 11-16-year-olds (adolescents). Information on child’s and parent’s ethnicity, as well as parent’s gender, were only used to describe the sample, whilst information on child’s age and gender were used as moderators of the factorial structure.

#### Pandemic Anxiety Scale - Parent report (PAS-P)

The PAS-P was developed as a modified version of the self-report PAS to capture child and adolescent pandemic-related anxiety. The PAS consists of 7 items rated on a 5-point Likert scale from 0 (‘strongly disagree’) to 4 (‘strongly agree’):

1. My child is worried that they will catch COVID-19.
2. My child is worried that friends and family will catch COVID-19.
3. My child is afraid to leave the house right now.
4. My child is worried they might transmit the infection to someone else.
5. My child is worried about missing school/work.
6. My child is worried about the amount of money we have coming in.
7. My child is worried about the long-term impact this will have on their job prospects and the economy.

There are two sub-scales reflecting disease specific (items 1–4) and consequence specific (items 5–7) anxieties related to the pandemic. The scale can be adjusted for other pandemic contexts by, for example, replacing the contextual cues for COVID-19 in the first two items with the relevant virus/disease. To yield a sub-scale score, the sub-scale item scores are summed, with larger scores representative of higher levels of that specific type of pandemic-related anxiety. The self-report PAS has been validated in adults and adolescents with acceptable internal consistency (α: 0.78–0.60) and discriminant validity (between factor *r* = .36), as well as moderate concurrent validity (*r*: 0.33-0.39) with other mental health symptom measures (McElroy et al., 2020).

#### Data analysis

The analysis plan was pre-registered on the Open Science Framework (OSF; https://osf.io/gx9sz)^2^. The data was cleaned in R (R Core Team, 2022). The longitudinal analysis was conducted in Mplus (Muthén & Muthén, 2017). Other factor analyses were conducted in R (R Core Team, 2022), using the *lavaan* (Rosseel, 2012), *EFATools* (Steiner & Grieder, 2020), and *psych* (Revelle, 2023) packages.

**Fig. 1:**
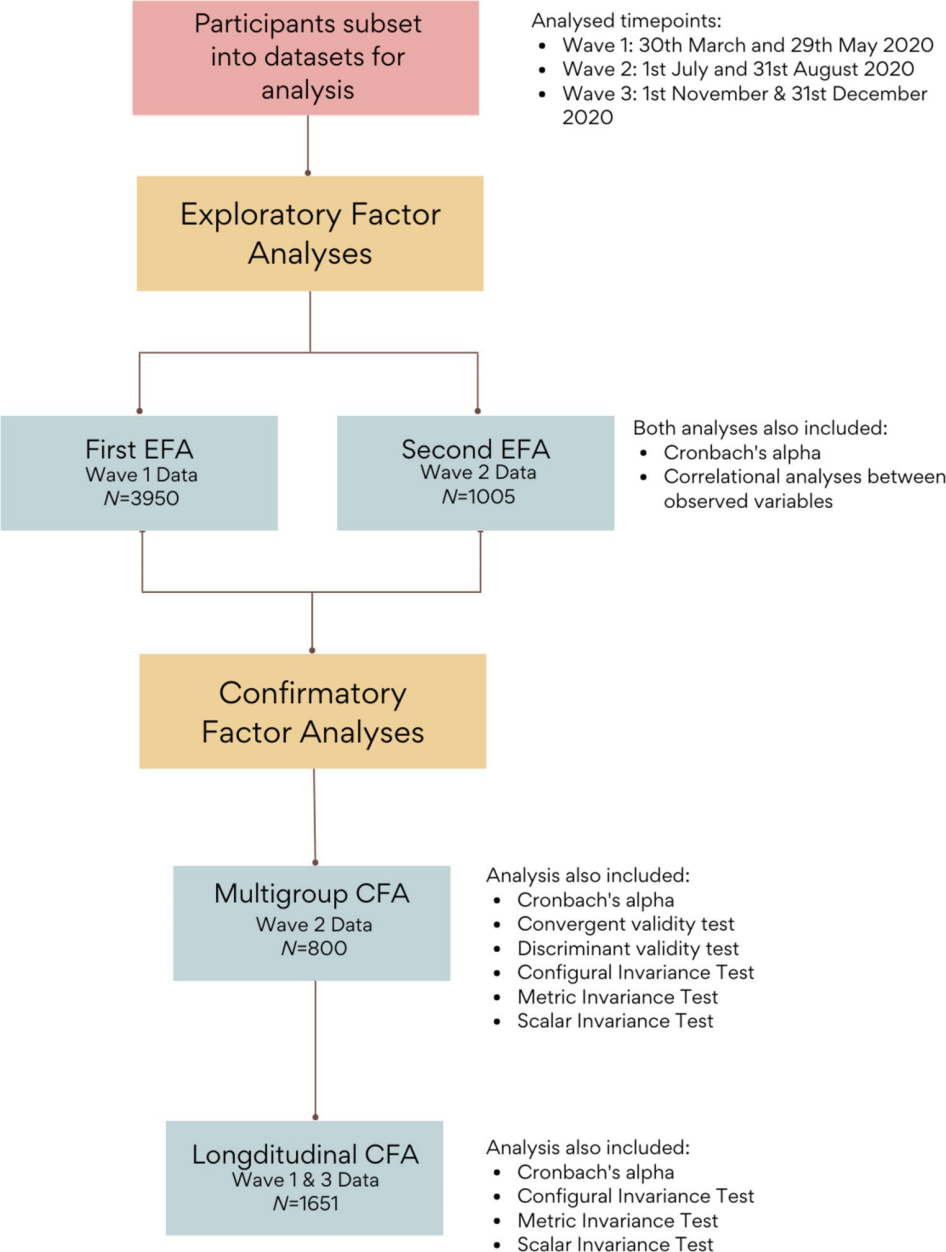
Outline of data sub-samples and the associated analyses

Four sub-samples of participants were used in the present work, with each sub-sample used in a different analysis (Fig. 1). Firstly, two exploratory factor analyses (EFAs) were conducted to assess latent structure using data from Wave 1 and Wave 2 separately as a form of cross-validation (Thompson, 2004). Demographic information for all sub-samples can be found in Table 1. Data were assessed for factor analysis suitability using the Kaiser-Meyer-Olkin Measure of Sampling Adequacy statistic (KMO > 0.65; Kaiser & Rice, 1974) and Bartlett’s test of sphericity (*p* < .05). Factor extraction was informed through a parallel analysis scree plot and the Kaiser-Guttman rule (eigen values > 1.00). Both EFAs used maximum likelihood (ML) estimation with goemin (oblique) rotation.

The factor structures derived from the EFAs were then validated using two confirmatory factor analysis (CFA) sub-samples. Specifically, a sub-sample of data from Wave 2 was used to assess convergent validity, discriminant validity, and measurement invariance based on child age and child gender (mCFA). Another independent sample with data from Wave 1 and 3 was used to assess measurement invariance over time using a longitudinal CFA. Convergent validity, referring to the internal consistency between subscales, that is the extent to which the subscales measure dissimilar constructs, in the mCFA was determined by factor loadings greater than 0.40 (Matsunaga, 2010), composite reliability equal or greater than 0.70 (Hair et al., 2011), and average variance extracted (AVE) equal or greater than 0.50 (Hair et al., 2011). Discriminant validity was accepted if AVE was greater than the squared correlation estimates between the same factors (Fornell & Larcker, 1981). Iterative model fit comparisons were used to assess existence of specific types of measurement invariance including configural invariance (i.e., varying factor loadings), metric invariance (i.e., equal factor loadings), and scalar invariance (i.e., equal intercepts) between groups and over time, respectively. Internal reliability of the PAS-P was assessed in all four sub-samples using Cronbach’s alpha.

The four different sub-samples of participants (Table 1) were selected based on the following procedure. The longitudinal CFA sample (*n* = 1651) was identified first. These participants were selected based on having responses to the PAS-P at Wave 1 and Wave 3. Any participants included in the longitudinal CFA had their data removed from the pool of potential data for other analyses. The sample for the multigroup CFA analyses (*n* = 800) was then identified from Wave 2 participations, with the data stratified by age (4–10 years; 11–16 years) and gender (girls; boys) to ensure sufficient sample sizes of each group (*n* > 100) in the invariance analysis; any participants included in the CFAs had their data removed from the pool of potential data for the EFA analyses. Finally, the rest of the participants’ data from Wave 1 and 2 were assigned to the first and second EFA samples (EFA1 *n* = 3950; EFA2 *n* = 1005), respectively. The two EFA samples included an overlap of 647 participants who took part in both waves. Based on rules-of-thumb for sample sizes (e.g., Comrey & Lee, 1992), the current research prespecified an absolute minimum of 200 participants for the EFA and single CFA analysis, and an absolute minimum of 500 participants for the longitudinal CFA. However, all available data was used from each wave to ensure sufficient power.

**Table 1:** Participant demographic data disaggregated by analysis sub-sample (*N* = 6,759)

		EFA1: Wave 1	EFA2: Wave 2	mCFA: Wave 2	lCFA: Wave 1 & 3
		(*n* = 3950)	(*n* = 1005)	(*n* = 800)	(*n* = 1651)
Age					
Mean *(SD)*		9.45 (3.53)	8.83 (3.14)	10.28 (2.50)	9.13 (3.39)
4–10 years		2468 (62.5%)	754 (75.0%)	400 (50.0%)	1111 (67.3%)
11–17 years		1482 (37.5%)	251 (25.0%)	400 (50.0%)	540 (32.7%)
Gender					
Boys		2048 (51.8%)	535 (53.2%)	400 (50.0%)	836 (50.6%)
Girls		1862 (47.1%)	449 (44.7%)	400 (50%)	815 (48.4%)
Other response/Prefer not to say		40 (1.0%)	21 (2.1%)	-	-

Participants whose self-identified gender was “Other response/Prefer not to say” were excluded from multigroup or longitudinal CFAs as these analyses assessed whether there were differences in responding between gendered groups

Model fit was evaluated using traditional global fixed fit cut-offs and dynamic fit cut-offs, as outlined in the pre-registration. Model fit indices are sensitive to complex interactions of data and model characteristics, such as sample size, degrees of freedom (Kenny et al., 2015), and model type (Fan & Sivo, 2007). Thus, traditional fixed cut-offs are not inherently generalizable (Hu & Bentler, 1998). Instead, model fit can be assessed through dynamic cut-offs derived using simulation techniques tailored to the specific model and analysed data characteristics (McNeish & Wolf, 2023). Global fit indices were assessed using the following cut-offs: RMSEA < 0.080, SRMR < 0.080, and CFI ≥ 0.950 (Kline, 2015). Dynamic fit cut-offs were simulated and tailored to the CFAs using R Shiny application version 1.1.0 (McNeish & Wolf, 2023). As per the pre-registration, complete success for CFAs would occur if all global goodness-of-fit index thresholds were met and partial success would occur when index thresholds were within 0.20 of those outlined. We used the following criteria of model fit change when comparing configural, metric, and scalar measurement invariance models: ΔCFI ≤ 0.01 (Chen, 2007; Cheung & Rensvold, 2002), ΔRMSEA>-0.020, metric invariance ΔSRMR>-0.030 and scalar invariance ΔSRMR>-0.015 (Chen, 2007).

## Results

### Exploratory factor analysis

Data from both EFA sub-samples were determined to be suitable for factor analysis. The KMO test for sampling adequacy was adequate, at 0.84 and 0.78 for the first and second sub-sample respectively (Kaiser, 1974). Bartlett’s test of sphericity was significant for both the EFA1 (χ^2^(21) = 8598.46, *p* < .001) and EFA2 (χ^2^(21) = 2391.79, *p* < .001) dataset, indicating suitable factorability of the correlation matrix (Field, 2013). No items in either dataset demonstrated skew (> 2.0) or kurtosis (> 2.0) and there were no outliers ± 3.5 SDs from the mean (Tabachnick & Fidell, 2007). Table 2 outlines the means and standard deviations for each item per sub-sample (see Table S2 and Figure S2 in Supporting Information for further details of data distribution).

**Fig. 2:**
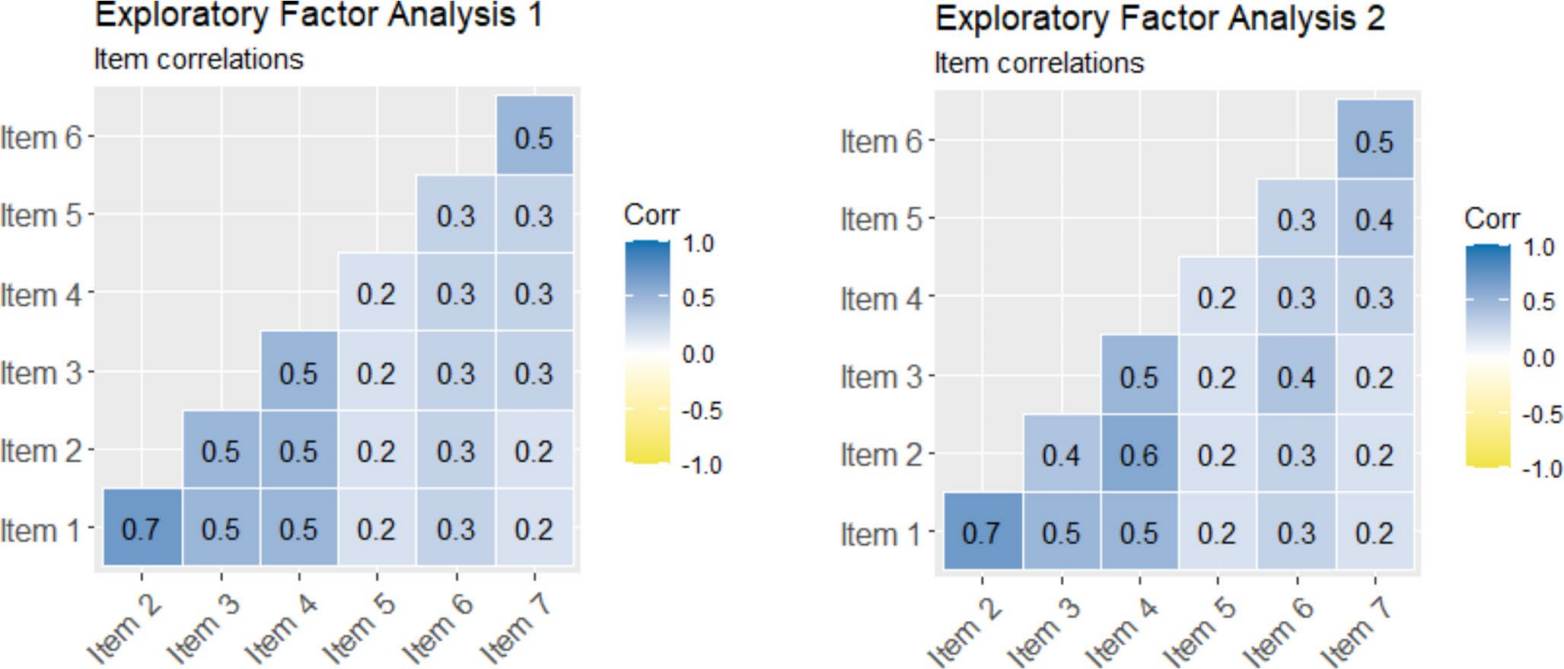
Correlations between PAS-P item values in EFA1 (left) and EFA2 (right) sub-samples

The EFAs on the data from both sub-samples produced highly similar two-factor solutions^3^ which corresponded to previous validations of the self-report PAS (e.g., McElroy et al., 2020), meeting partial success criteria for EFA1 (as one item did not load on to the pre-stipulated factor at the outlined threshold) and full success criteria for EFA2. Indeed, the appropriate items loaded on to the theoretically stipulated factors of Disease Anxiety (Factor 1) and Consequence Anxiety (Factor 2) that explained 60–61% and 39–40% of variance, respectively. Most factor loadings were similar between the two sub-samples (Table 3), except for < 0.40 loading of item 5 (*“My child is worried about missing school/work”*) in EFA1 during the first lockdown than in EFA2. Yet, due to the acceptable loading of this item in EFA2, when restrictions were easing, item 5 was retained for the subsequent analyses. The correlation between the latent factors was 0.44 in EFA1 and 0.38 in EFA2 (see Fig. 2 for correlations between items) indicating that factors were sufficiently distinct (< 0.85, Brown, 2015). Internal consistency, as indicated by Cronbach’s alpha, was acceptable (Taber, 2018) and ranged from 0.62 to 0.82 for EFA1 sub-sample and 0.66-0.82 for EFA2 sub-sample.

**Table 2:** Means (SDs) for PAS-P items and subscales for each sub-sample

	EFA1: Wave 1 (*n*=3950)	EFA2: Wave 2 (*n*=1005)	mCFA: Wave 2					lCFA:	
			Total (*n*=800)	Age		Gender		Wave 1 (*n*=1651)	Wave 3 (*n*=1651)
				4-10 years (*n*=400)	11-16 years (*n*=400)	Boys (*n*=400)	Girls (*n*=400)		
1.	1.90 (1.06)	1.87 (1.07)	1.90 (1.09)	1.84 (1.08)	1.97 (1.09)	1.85 (1.07)	1.96 (1.10)	1.83 (1.06)	1.85 (1.02)
2.	2.30 (1.09)	2.19 (1.11)	2.25 (1.12)	2.17 (1.14)	2.34 (1.10)	2.20 (1.14)	2.31 (1.10)	2.28 (1.13)	2.24 (1.08)
3.	1.30 (1.10)	0.91 (0.99)	0.99 (1.05)	0.84 (0.97)	1.14 (1.09)	0.98 (1.052)	1.01 (1.04)	1.17 (1.08)	0.66 (0.85)
4.	1.46 (1.04)	1.35 (1.10)	1.48 (1.15)	1.27 (1.08)	1.69 (1.17)	1.47 (1.16)	1.50 (1.13)	1.35 (1.04)	1.53 (1.13)
5.	1.64 (1.30)	1.34 (1.31)	1.56 (1.30)	1.34 (1.26)	1.79 (1.31)	1.42 (1.31)	1.71 (1.28)	1.65 (1.33)	1.27 (1.25)
6.	1.02 (1.03)	0.83 (0.96)	0.91 (1.03)	0.71 (0.92)	1.10 (1.09)	0.91 (1.06)	0.91 (0.99)	0.87 (1.02)	0.78 (0.95)
7.	0.90 (1.04)	0.75 (0.99)	1.02 (1.12)	0.53 (0.78)	1.50 (1.19)	1.02 (1.18)	1.02 (1.05)	0.75 (1.00)	0.94 (1.10)
Disease Anxiety	6.96 (3.45)	6.33 (3.49)	6.63 (3.65)	6.13 (3.49)	7.14 (3.73)	6.50 (3.69)	6.77 (3.60)	6.63 (3.47)	6.28 (3.32)
Consequence Anxiety	3.56 (2.57)	2.92 (2.54)	3.49 (2.70)	2.58 (2.31)	4.39 (2.77)	3.34 (2.85)	3.63 (2.55)	3.27 (2.49)	2.99 (2.63)

**Table 3:** Factor loadings for items on the PAS-P across the two sub-samples

	EFA1 (*n* = 3950)		EFA2 (*n* = 1005)	
	Factor 1	Factor 2	Factor 1	Factor 2
1.	0.88		0.89	
2.	0.80		0.85	
3.	0.55		0.52	
4.	0.49		0.59	
5.		0.38^a^		0.47
6.		0.67		0.67
7.		0.78		0.78

^a^Below loading threshold of 0.40

### Confirmatory factor analysis

We assessed the two-factor structure identified as the preferred model in the EFAs using the total multigroup CFA sub-sample (*n* = 800) from the period of easing restrictions. Means and standard deviations for PAS-P items for this sub-sample can be found in Table 2. In addition to global fit indices, the following dynamic indices were used to assess model fit for this analysis: RMSEA = 0.10, SRMR = 0.06, and CFI = 0.96. Based on the dynamic and global fit indices, the two-factor CFA model met the predefined criteria for the partial success (RMSEA = 0.106, SRMR = 0.056, and CFI = 0.940) and fit the data significantly better than a single latent factor model (RMSEA = 0.205, SRMR = 0.127, and CFI = 0.767), Δχ(1) = 353.28, *p* < .001. The internal reliability for both sub-scales indicated by two factors was also satisfactory at Cronbach’s α = 0.85 for Disease Anxiety, and α = 0.68 for Consequence Anxiety.

**Fig. 3:**
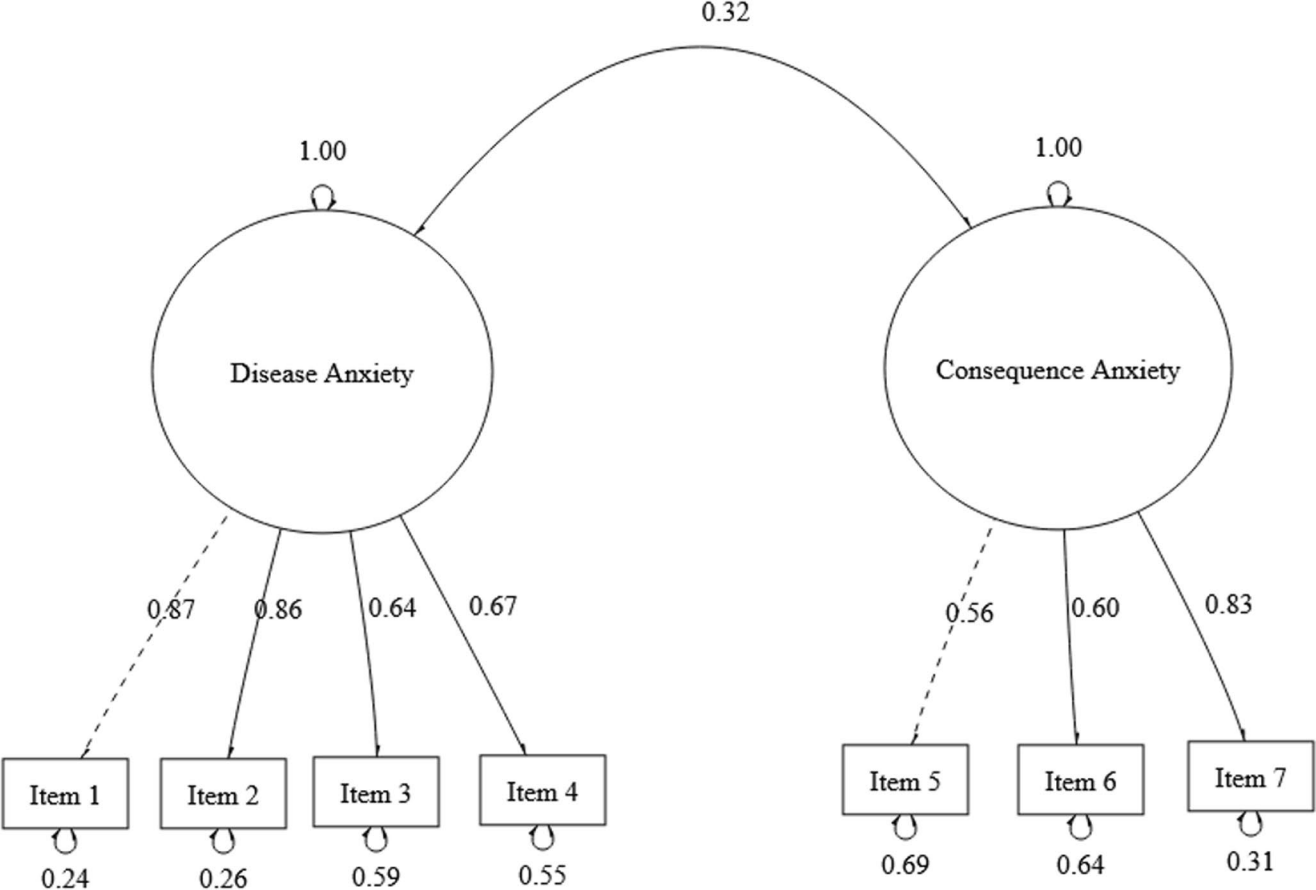
Factor structure and loadings for the two-factor model of the parent report pandemic anxiety scale

The two-factor model was subject to tests of convergent and discriminant validity, which indicated that the sub-scales might be measuring overlapping concepts but that they were unrelated. Specifically, convergent validity refers to the degree to which scale items are measuring the same concept or construct (Cunningham et al., 2001). Factor loadings for both latent factors were greater than 0.40, ranging from 0.56 to 0.87 (Fig. 3). Composite reality fell below 0.70 for both Disease (0.66) and Consequence (0.43) Anxiety sub-scales. However, only the AVE for Disease Anxiety (0.59), but not Consequence Anxiety (0.45) was above the threshold of over 0.50. Discriminant validity tests whether concepts which are not supposed to be related are indeed unrelated. The AVE for both Disease Anxiety (0.59) and Consequence Anxiety (0.45) were greater than the squared correlation matrix value between the two factors (0.10), suggesting that each factor shared more variance with its relevant observed variables than with the other observed variables.

### Multigroup confirmatory factor analysis

The invariance of factor structure across gender groups (girls and boys) and age groups (4–10 years and 11–16 years) was assessed by constructing increasingly restricted models. The fit indices for each model can be found in Table 4. The configural invariance models allowed model parameter estimates to vary across groups without imposing equality constraints, the metric invariance models constrained factor loadings to be equal across groups, and the scalar invariance models then constrained intercepts to be equal across groups. For gender, dynamic fit indices achieved adequate fit and change in goodness-of-fit indices did not indicate iterative worsening in model fit across the configural, metric, and scalar invariance models. Hence, the measurement invariance held across gender groups with two-factor structure, factor loadings, and intercepts of items equivalent across boys and girls.

For age, the configural, metric, and scalar invariance models also all achieved acceptable to good dynamic model fit. However, the change in goodness-of-fit indices indicated significant worsening in model fit between metric to scalar invariance models, suggesting that item thresholds did differ across groups (i.e., non-invariance). According to further examination, items 4 (“*My child is worried they might transmit the infection to someone else”*) and 7 (*“My child is worried about the long-term impact this will have on their job prospects and the* *economy*”) had the biggest impact on model fit. Higher scores were found on both items for 11-16-year-old children/young people (Item 4: *M* = 1.69, *SD* = 1.15; Item 7: *M* = 1.50, *SD* = 1.11) than for 4-10-year-old children (Item 4: *M* = 1.27, *SD* = 1.08; Item 7: *M* = 0.53, *SD* = 0.78). The scalar invariance model for age was, thus, re-examined without imposing intercept equality constraints for these items. This resulted in acceptable model fit indices and their change indicating partial invariance in responses with similar two-factor structure, factor loadings, and most item intercepts equivalent across age groups.

**Table 4:** Goodness-of-fit indices for multigroup and longitudinal CFA models

Model		CFI	∆CFI	RMSEA	∆RMSEA	SRMR	∆SRMR
Gender Invariance							
Configural Model		0.942	-	0.106	-	0.052	-
Metric Model		0.939	0.003	0.100	0.006	0.057	− 0.005
Scalar Model		0.935	0.004	0.095	0.005	0.060	− 0.003
Age Invariance							
Configural Model		0.955	-	0.092	-	0.046	-
Metric Model		0.950	0.005	0.089	0.003	0.051	− 0.005
Scalar Model		0.926	0.024	0.100	− 0.011	0.070	− 0.019
Partial Scalar Model ^a^		0.945	0.005	0.089	< 0.001	0.053	− 0.002
Longitudinal Invariance							
Configural Model		0.948	-	0.104	-	0.047	-
Metric Model		0.966	− 0.018	0.082	0.022	0.044	0.003
Scalar Model		0.922	0.044	0.105	− 0.023	0.048	− 0.004
Partial Scalar Model ^b^		0.947	0.019	0.089	− 0.007	0.046	− 0.002

^a^Intercepts for items 4 and 7 were allowed to vary between groups. ^b^Intercept for item 3 was allowed to vary between time points. More constrained models are accepted if ΔCFI ≤ 0.01, ΔRMSEA>-0.020, metric invariance ΔSRMR>-0.030, and scalar invariance ΔSRMR>-0.015

### Longitudinal confirmatory factor analysis

Participants’ responses to the PAS-P at two repeated measures (*n* = 1651; Table 2) of Wave 1 (during the first lockdown) and Wave 3 (when restrictions were reintroduced) were used to assess measurement invariance of the two-factor structure over time. Cronbach’s alpha statistics for the longitudinal dataset found acceptable levels of internal consistency (α: 0.75–0.85).

The fit indices for each of the four measurement invariance models (configural, metric, scalar, partial scalar) are shown in Table 4. The configural model, which allowed factor loadings to vary between time points, demonstrated poor model fit for CFI and RMSEA indices^4^. The metric model, which constrained factor loadings to be equal across time points, achieved good model fit and significantly improved model fit in comparison to the configural invariance model. However, the scalar invariance constraints on item intercepts significantly worsened the model fit. When the most impactful item 3 (“*My child is afraid to leave the house right now*”) was allowed to vary between the time points, partial scalar invariance model still fit the data worse, according to change in CFI, than the metric invariance model but better than the configural invariance model. Given that the difference from the metric model was likely driven by the initial model improvement, we consider that partial scalar invariance held longitudinally with acceptably similar two-factor structure, factor loadings, and intercepts of items between first national lockdown (Wave 1) and later reintroduction of restrictions (Wave 3).

## Discussion

The present study aimed to validate the parent-report version of the PAS-P, a measure of children and young people’s pandemic-related anxiety. The analyses showed that the PAS-P had (a) good internal consistency as indicated by Cronbach’s alpha, (b) a two-factor structure for Disease Anxiety and Consequence Anxiety, (c) adequate factor solution discriminant (but not convergent) validity, and (d) at least partial invariance in latent construct measurement across child age, gender, and time. Taken together, our results suggest that the PAS-P is a generally reliable and valid measure of pandemic-related anxiety in children as reported by parents, however, measuring potentially overlapping concepts.

In general, our findings extend the use of the PAS to parent report but are otherwise comparable with the original self-report PAS (McElroy et al., 2020) validated in adults and adolescents. As in previous studies, we found that a two-factor structure underpinned the measure reflecting the two components of major concern about pandemics: (a) Disease Anxiety with concerns about catching and transmitting the virus and (b) Consequence Anxiety with concerns about the consequences of the pandemic (e.g., missing school or work). We also expanded on the approach taken in previous evaluations of the PAS by demonstrating that the two-factor structure, factor loadings, and intercepts were at least partially invariant across time. This indicates that the PAS-P structure is generally robust in the face of varying contextual factors such as changing regulations and restrictions.

Whilst full metric invariance held over time, only partial scalar invariance was achieved indicating inconsistency in the relationship between item means and level of disease and consequence anxiety across the first UK national lockdown and later that year when restrictions were reintroduced. Levels of worry about leaving the home (item 3) particularly decreased from March-May (the first national lockdown where children were largely home-schooled) to November-December 2020 (where there was a tightening of restrictions, but schools remained largely open). Amongst other potential explanations, this decrease may reflect that children and young people were generally attending school face-to-face and were more freely able to leave the house during this period. It may also be possible that the documented reduced concerns relating to the virus may be due to normalisation of reduced restrictions from the summer period (Williams & Dienes, 2021). Full scalar invariance is rare in empirical research (Robitzsch & Lüdtke, 2023). Therefore, minor parameter adjustments are acceptable to achieve partial invariance (Putnick & Bornstein, 2016) and is unlikely to undermine the PAS-P usability for overtime or group comparisons. Yet, the change in scores across time is interesting as it documents how the perceived consequences of the virus changed and may be sensitive to both population and participant level variables.

The multigroup invariance testing indicated that the PAS-P is also reliable and valid for use with children (4-10-year-olds) and adolescents (11-16-year-olds), as well as across girls and boys. Full scalar invariance was achieved for gender with similar factor structure, factor loadings, and means between boys and girls. Partial scalar invariance with similar factor structure and loadings was also achieved across age groups. However, there were some differences in the relationship between item response means and latent construct means according to age. Specifically, parents reported adolescents having higher levels of anxiety about COVID-19 risk of transmission to others (item 4) and long-term impact on their job prospects and the economy (item 7) than children. This is likely to reflect both developmental differences and qualitatively different pandemic experiences across the age groups. For instance, recent qualitative research showed that worries about the future during the pandemic noticeably varied by age, with older adolescents having concerns about the impact on their GCSE exams and preparation for their future, whilst younger children worried more broadly about not being able to attend school (Pearcey et al., 2023). Furthermore, ability to grasp abstract concepts substantially improves between ages 10 and 12 (e.g., Caramelli et al., 2004) which may result in higher distress and concern about the social, moral, emotional, and economic risks and consequences in adolescents in comparison to children.

Whilst our findings are generally comparable with the previous PAS self-report validation (McElroy et al., 2020), a couple of noteworthy differences have occurred. Firstly, the composite reliability and AVE thresholds (statistics measuring internal consistency between the subscales) in our study indicated that the subscales may measure overlapping latent variables. As neither AVE or composite reliability were directly investigated in the self-report PAS (McElroy et al., 2020), it suggests either that the originally proposed constructs of disease and consequence anxiety overlap, or that current participants have interpreted the items measuring different constructs in a similar way. Hence, we are unable to conclude that the items for each factor measure distinct and unique constructs. Secondly, the CFA analysis in the current study produced a greater range of loading values (0.43–0.87) compared to McElroy et al. (2020) study (0.51–0.82). It is likely that this reflects differences in sample sizes between the two studies. McElroy et al. (2020) conducted a single CFA on a sample of 2,426 participants and the present study involved multiple CFAs on multiple samples with 1,651 and 800 participants. In factor analyses, greater sample sizes reduce error, meaning that the yielded solution is more stable and reliably reflects the factorial structure of the population (MacCallum et al., 1999). The smaller sample sizes used in the present research may therefore be subject to greater error, leading to greater variation in loading values. The factor loadings in the current work were, however, above the pre-established acceptable threshold and in line with previous work. As such, our validation work provides evidence of the strong psychometric properties of the PAS-P.

Strengths of this work include the large sample size, high degrees of independence between samples, and comprehensive validation analyses with consistent findings across multiple EFA and CFA models. Nevertheless, there are some limitations including the convenience sample which may introduce selection bias and whose responses are not necessarily representative of the general population of the UK. Indeed, participating parents self-identified as predominantly female and white, which may further reduce the generalisability of the results. Future psychometric evaluations of PAS-P should be conducted in more diverse samples to ensure external validity of the scale. It is also worth noting that the longitudinal CFA model did not meet the predefined fit criteria for the configural model. Upon further investigation, this issue occurred due to the poor fit of the Wave 1 data. Similarly, the EFA using different Wave 1 data indicated poorer loading of the item measuring concerns about missing school/work, although it otherwise confirmed the two-factor structure as appropriate during the first national lockdown. This was resolved when the longitudinal model factor loadings were constrained overtime indicating that they did not significantly differ between Wave 1 and 3. As with all structural equation modelling, models other than those specified may exist that fit the data at approximately the same or increased level of goodness-of-fit. As such, while the available evidence suggests that a two-factor structure is an appropriate model, conclusions should be reasonably tempered to reflect that the model discussed above is only plausible, although well supported.

### Future directions and clinical implications

Further research is warranted to confirm and advance the PAS-P validity and generalisability. To further validate the measure, future conceptual replication work should cross-validate the factor structure and validity (e.g., construct validity) of PAS-P responses to further confirm the underlying model and evaluate whether other sample characteristics (e.g., pre-existing anxiety problems or health conditions), as well as contextual or boundary conditions (e.g., future or different pathogen/viral pandemics) influence responses or psychometric properties. For instance, translation and psychometric evaluation of this scale should be carried out to determine its suitability and validity for the use in different languages and cultures. Further examination of convergent validity is also needed for the use of both PAS and PAS-P to confirm that disease and consequence anxiety sub-scales measure meaningfully distinct concepts.

The current evidence for PAS-P reliability, validity, and measurement invariance have implications for clinicians and researchers aiming to address the mental health impacts of the pandemics. For example, the current study supports parent-reported PAS as a reliable assessment of pandemic-related worries in pre-adolescent children, who may not be able to reliably self-report on their own. For those working with adolescents, this provides an opportunity for a more comprehensive assessment of their pandemic-related worries by enabling triangulation between nuances in young people’s subjective experiences of anxiety captured by self-reported PAS and parental observations via PAS-P. Furthermore, it allows for a better understanding and addressing of potential processes underlying changes in and trajectories of children and young people’s mental health throughout the pandemic (Creswell et al., 2021b; Guzman Holst et al., 2023). For instance, current findings suggest that worries about leaving the house may have been particularly prominent during the early pandemic, whilst worries about disease transmission and economic consequences may explain some of the age differences observed in responsivity to the pandemic restrictions. This provides potential targets for future intervention studies aiming to support mental health needs of children and young people during pandemics or other public health crises. The current study confirms that PAS measure can be reliably used to explore the longitudinal trajectories of pandemic-related anxieties over time, how it links with mental health symptoms, and examine how interventions or external factors may influence them.

## Conclusions

In conclusion, the findings demonstrate that the PAS-P is a robust parent/carer report measure of two distinct forms of pandemic-related anxiety (disease anxiety and consequence anxiety), suitable for reporting on 4-16-year-old children. This measure could be used to identify children and young people who are experiencing elevated levels of anxiety during and in response to pandemics and, also, to develop and evaluate interventions for children that address disease or consequence anxieties related to pandemics. Further conceptual replications which validate the PAS-P are encouraged in the future.

## Electronic supplementary material

Below is the link to the electronic supplementary material.

Supplementary Material 1

## Data Availability

The full data that support the findings of this study are available on request from the corresponding author due to privacy restrictions. The Co-SPACE data are partially available under safeguarded access via the UK Data Service at http://doi.org/10.5255/UKDA-SN-8900-1, reference number SN 8900. Research materials for the Co-SPACE project can be found on the Open Science Framework: https://osf.io/8zx2y/.

## Footnotes

1 The school term in England commenced in September, outside of the time period of interest.

2 The analysis reported herein differed slightly from the pre-registration. Firstly, only the items retained in McElroy and colleagues’ (2020) validated PAS (*n* = 7) were included, as the original 9-item version did not fit the theorised. Secondly, in the EFA analyses, cross-loading items < 0.40 were retained. Thirdly, no variables were suppressed as all loaded near or above > 0.40 for both analyses (Stevens, 1992). Finally, to maintain consistency across all invariance testing in the present report, scalar invariance was calculated for the longitudinal CFA, too.

3 Based on the extraction of one eigenvalue > 1.00 and the point of inflexion potentially falling at the second or third factor, parallel analysis indicated two- or three-factor solution was suitable for the EFA1 sub-sample. Both of these factor solutions, thus, were further explored. As only one variable loaded at a value of ≥ 0.40 on the third factor (Figure S3 and Table S3 in Supporting Information), a two-factor model was therefore preferred.

4 The reasons for unsatisfactory configural fit were further investigated by conducting two separate CFAs for each wave. In addition to global fit indices, the following dynamic indices were used for Wave 1 model fit: RMSEA = 0.053, SRMR = 0.028, and CFI = 0.986; and Wave 3: RMSEA = 0.155, SRMR = 0.082, and CFI = 0.891. Model goodness-of-fit was poor for Wave 1 (RMSEA = 0.100, SRMR = 0.046, and CFI = 0.939), but acceptable for Wave 3 (RMSEA = 0.114, SRMR = 0.060, and CFI = 0.931).
