# Supplementary material for: Validation of the Parent-report Pandemic Anxiety Scale (PAS-P) in the context of COVID-19
Source: Curr Psychol. 2024 Oct 10;43(42):32539–51. doi: 10.1007/s12144-024-06784-x (PMC11611924; doi:10.1007/s12144-024-06784-x)
Supplement: Supplementary file 1 — Supplementary Material 1 [file 12144_2024_6784_MOESM1_ESM.docx]

**Table S1**

*Participants’ Demographic Data (N=6,759)*

| Group | | Parents | |  | Children | |
| --- | --- | --- | --- | --- | --- | --- |
|  |  | *n* | % |  | *n* | % |
| Gender | |  |  |  |  |  |
|  | Women/Girls | 6253 | 92.5% |  | 3251 | 48.1% |
|  | Men/Boys | 459 | 6.8% |  | 3464 | 51.3% |
|  | Other response/Prefer not to say | 47 | 0.7% |  | 44 | 0.7% |
| Ethnicity | |  |  |  |  |  |
|  | White | 6339 | 93.8% |  | 6435 | 95.2% |
|  | Asian | 118 | 1.8% |  | 99 | 1.5% |
|  | Black | 33 | 0.5% |  | 27 | 0.4% |
|  | Middle Eastern | 24 | 0.4% |  | 19 | 0.3% |
|  | Mixed race | 129 | 1.9% |  | 86 | 1.3% |
|  | Other ethnic group | 56 | 0.8% |  | 35 | 0.5% |
|  | Prefer not to say | 63 | 0.9% |  | 58 | 0.9% |
| Age | |  |  |  |  |  |
|  | 4-10 years | -- | -- |  | 4299 | 63.6% |
|  | 11-16 years | -- | -- |  | 2460 | 36.4% |

**Figure S1**

*Timeline of significant events which occurred during the timepoints analysed (Waves 1, 2 and 3).*


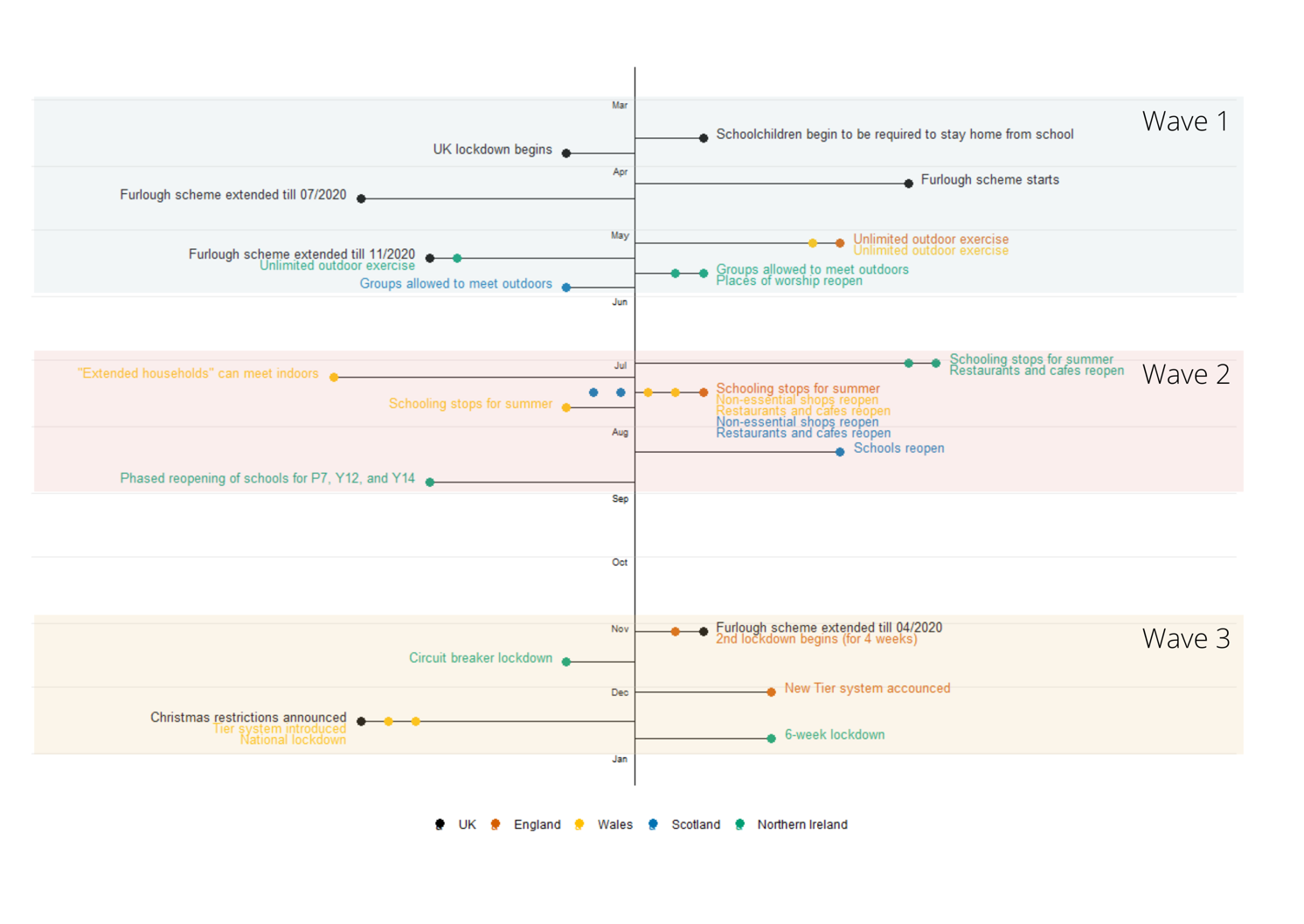


**Table S2**

*Summary Statistics per PAS item per sub-sample*

| PAS item | Original PAS item | Item wording | EFA1 (n=3950) | | | | |  | EFA2 (n=1005) | | | | |
| --- | --- | --- | --- | --- | --- | --- | --- | --- | --- | --- | --- | --- | --- |
|  |  |  | *M* | *SD* | Median | Skew | Kurtosis |  | *M* | *SD* | Median | Skew | Kurtosis |
| Item 1 | 2 | My child is worried that they will catch COVID-19 | 1.90 | 1.06 | 2 | 0.17 | -0.64 |  | 1.87 | 1.07 | 2 | 0.16 | -0.73 |
| Item 2 | 3 | My child is worried that family and friends will catch COVID-19 | 2.30 | 1.09 | 2 | -0.27 | -0.72 |  | 2.19 | 1.11 | 2 | -0.15 | -0.80 |
| Item 3 | 4 | My child is afraid to leave the house right now | 1.30 | 1.10 | 1 | 0.80 | -0.02 |  | 0.91 | 0.99 | 1 | 1.14 | 0.81 |
| Item 4 | 5 | My child worried they might transmit the infection to someone else | 1.46 | 1.04 | 1 | 0.40 | -0.48 |  | 1.35 | 1.10 | 1 | 0.53 | -0.59 |
| Item 5 | 7 | My child is worried about missing school/work | 1.64 | 1.30 | 1 | 0.27 | -1.17 |  | 1.34 | 1.31 | 1 | 0.60 | -0.90 |
| Item 6 | 8 | My child is worried about the amount of money we have coming in | 1.02 | 1.03 | 1 | 0.91 | 0.22 |  | 0.83 | 0.96 | 1 | 1.04 | 0.37 |
| Item 7 | 9 | My child is worried about the long-term impact this will have on their job prospects and the economy | 0.90 | 1.04 | 1 | 1.03 | 0.32 |  | 0.75 | 0.99 | 0 | 1.25 | 0.93 |

**Figure S2**

*PAS item score distribution for a) EFA1 and b)* *EFA2 sub-sample*

a)
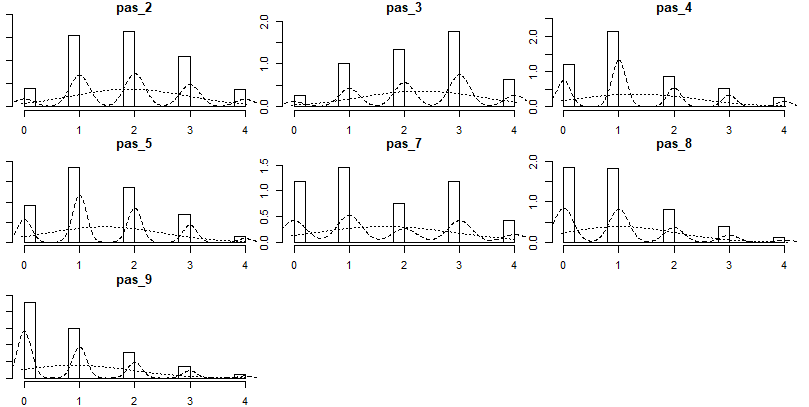


b)
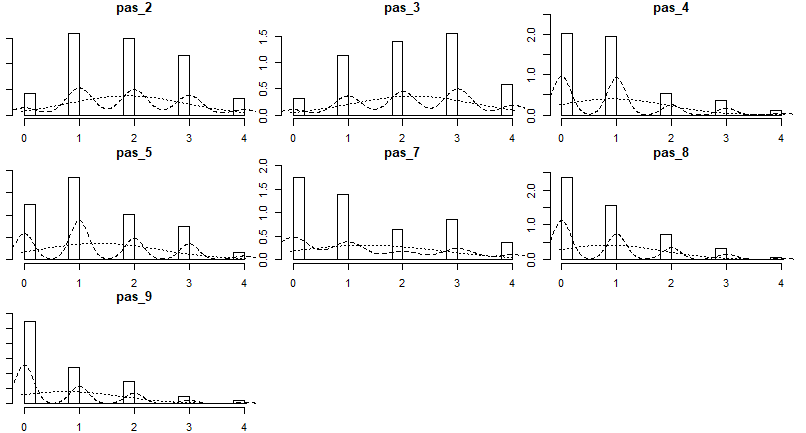


*Note.* Item names indicate numberingin the original PAS scale, see Table S1 for corresponding item wording.

**Figure S3**

*Parallel Analysis Scree Plots for a) EFA1 and b) EFA2 sub-sample*

*a)*
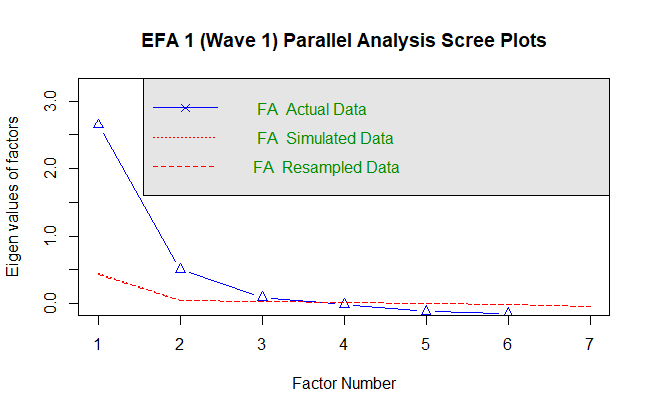


*b)*
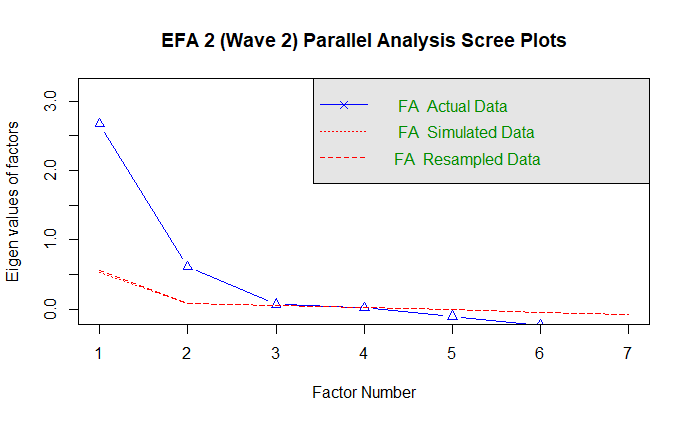


**Table S3**

*Factor loadings for a two- and three-factor solution per EFA sub-sample*

|  |  | Two-factor structure | | | | |  | Three-factor structure | | | | | |
| --- | --- | --- | --- | --- | --- | --- | --- | --- | --- | --- | --- | --- | --- |
|  |  | Factor loadings | | Communality | Uniqueness | Complexity |  | Factor loadings | | | Communality | Uniqueness | Complexity |
|  |  | 1^st^ | 2^nd^ |  |  |  |  | 1^st^ | 2^nd^ | 3^rd^ |  |  |  |
| EFA1 | |  |  |  |  |  |  |  |  |  |  |  |  |
|  | Item 1 | 0.88 |  | 0.74 | 0.26 | 1.01 |  | 0.70 |  | 0.16 | 0.64 | 0.36 | 1.10 |
|  | Item 2 | 0.80 |  | 0.64 | 0.36 | 1.00 |  | 0.91 |  |  | 0.79 | 0.21 | 1.01 |
|  | Item 3 | 0.55 | 0.19 | 0.43 | 0.57 | 1.23 |  |  |  | 0.99 | 1.00 | 0.01 | 1.00 |
|  | Item 4 | 0.49 | 0.26 | 0.42 | 0.59 | 1.52 |  | 0.36 | 0.23 | 0.21 | 0.41 | 0.59 | 2.32 |
|  | Item 5 | 0.09 | 0.38 | 0.18 | 0.82 | 1.10 |  | 0.13 | 0.40 |  | 0.19 | 0.81 | 1.28 |
|  | Item 6 | 0.07 | 0.67 | 0.49 | 0.51 | 1.02 |  |  | 0.65 | 0.06 | 0.48 | 0.52 | 1.02 |
|  | Item 7 |  | 0.78 | 0.58 | 0.42 | 1.01 |  |  | 0.80 |  | 0.61 | 0.40 | 1.01 |
| EFA2 | |  |  |  |  |  |  |  |  |  |  |  |  |
|  | Item 1 | 0.89 |  | 0.77 | 0.23 | 1.00 |  | 0.75 |  | 0.13 | 0.68 | 0.32 | 1.06 |
|  | Item 2 | 0.85 |  | 0.71 | 0.29 | 1.00 |  | 0.96 |  |  | 0.84 | 0.16 | 1.01 |
|  | Item 3 | 0.52 | 0.20 | 0.39 | 0.61 | 1.28 |  |  |  | 0.99 | 1.00 | 0.01 | 1.00 |
|  | Item 4 | 0.59 | 0.16 | 0.45 | 0.55 | 1.15 |  | 0.49 | 0.13 | 0.18 | 0.45 | 0.55 | 1.43 |
|  | Item 5 |  | 0.47 | 0.57 | 0.43 | 1.01 |  | 0.08 | 0.47 |  | 0.24 | 0.76 | 1.06 |
|  | Item 6 | 0.09 | 0.67 | 0.50 | 0.50 | 1.04 |  |  | 0.62 | 0.12 | 0.47 | 0.53 | 1.08 |
|  | Item 7 |  | 0.78 | 0.57 | 0.43 | 1.01 |  |  | 0.81 |  | 0.63 | 0.38 | 1.01 |
